# Neuroprotection Against MPP^+^-Induced Cytotoxicity Through the Activation of PI3-K/Akt/GSK3β/MEF2D Signaling Pathway by Rhynchophylline, the Major Tetracyclic Oxindole Alkaloid Isolated From *Uncaria rhynchophylla*

**DOI:** 10.3389/fphar.2018.00768

**Published:** 2018-07-19

**Authors:** Shengquan Hu, Shinghung Mak, Xialin Zuo, Haitao Li, Yuqiang Wang, Yifan Han

**Affiliations:** ^1^State Key Laboratory of Chinese Medicine and Molecular Pharmacology (Incubation), The Hong Kong Polytechnic University Shenzhen Research Institute, Shenzhen, China; ^2^Department of Applied Biology and Chemical Technology, Institute of Modern Chinese Medicine, The Hong Kong Polytechnic University, Hung Hom, Hong Kong; ^3^Institute of New Drug Research and Guangzhou Key Laboratory of Innovative Chemical Drug Research in Cardio-Cerebrovascular Diseases, Jinan University College of Pharmacy, Guangzhou, China

**Keywords:** Parkinson’s disease, MPP^+^, rhynchophylline, neuroprotection, MEF2D, GSK3β

## Abstract

Rhynchophylline is a major tetracyclic oxindole alkaloid in *Uncaria rhynchophylla*, which has been extensively used as traditional herb medicine for the prevention of convulsions and hypertension. However, there is still little evidence about the neuroprotective effects of rhynchophylline for Parkinson’s disease (PD), a neurodegenerative condition currently without any effective cure. In this present study, the neuroprotective molecular mechanisms of rhynchophylline were investigated in a cellular model associated with PD. It is shown that rhynchophylline (10–50 μM) greatly prevented neurotoxicity caused by 1-methyl-4-phenylpyridinium ion (MPP^+^) in primary cerebellar granule neurons, as evidenced by the promotion of cell viability as well as the reversal of dysregulated protein expression of Bax/Bcl-2 ratio. Very encouragingly, we found that rhynchophylline markedly enhanced the activity of the transcription factor myocyte enhancer factor 2D (MEF2D) at both basal and pathological conditions using luciferase reporter gene assay, and reversed the inhibition of MEF2D caused by MPP^+^. Additionally, pharmacological inhibition of PI3-Kinase or short hairpin RNA-mediated gene down-regulation of MEF2D abrogated the protection provided by rhynchophylline. Furthermore, Western blot analysis revealed that rhynchophylline could potentiate PI3-K/Akt to attenuate GSK3β (the MEF2D inhibitor) in response to MPP^+^ insult. In conclusion, rhynchophylline inhibits MPP^+^-triggered neurotoxicity by stimulating MEF2D via activating PI3-K/Akt/GSK3β cascade. Rhynchophylline is served as a novel MEF2D enhancer and might be a potential candidate for further preclinical study in the prevention of PD.

## Introduction

Parkinson’s disease is a severe neurodegeneration that damages the motor functions of patients (Kang et al., 2017). At cellular levels, PD is pathologically characterized by the selective and progressive dopaminergic neuronal death in the substantial nigra pars compacta (Martinez et al., 2017). Unfortunately, there is currently no curable treatment for PD. Current pharmacological interventions including monoamine oxidase-B inhibitors and precursor of dopamine could improve clinical symptoms but bring no potential disease-modifying benefits, largely because these drugs seldom alleviate dopaminergic neuronal loss. Therefore, the identification of neuroprotectants that could reverse the neuronal loss caused genetically or environmentally represents a novel direction for the development of anti-PD drugs (Guo et al., 2017).

Myocyte enhancer factor 2 protein consists of four distinct vertebrate isoforms (MEF2A-2D) and serves as a family of transcription factors responsible initially for muscle gene expression. Recent studies highlighted the distribution of MEF2 isoforms in the central nervous system. Over the past few years, extensive evidence, including the clinical findings that MEF2 levels were robustly decreased in postmortem brain of Parkinson’s patients (She et al., 2011), has suggested that inhibition of MEF2, and MEF2D in particular, is neuropathologically linked to PD (Yang et al., 2009), Moreover, MEF2D plays a critical role in neuronal survival in various cellular and animal experimental models related to PD (Mount et al., 2013; Hu et al., 2015b; Guo et al., 2017). In these experiment paradigms, MEF2 proteins serve as endpoints for multiple signaling cascades and thereby confer signal-responsiveness to downstream target genes. Consequently, activation of MEF2D is a significant mechanism in attenuating neurotoxicity such as damage to dopaminergic cells observed after exposure to PD-related toxins. For instance, activation of MEF2D by dimeric bis(3)-cognitin (Yao et al., 2012) and SU4312 (an anti-cancer agent) (Guo et al., 2017) protected dopaminergic neurons and attenuated Parkinsonian motor defects. These results strongly suggest that MEF2D represents a genuine neuroprotective target for the prevention and treatment of PD.

The dried stem and hook of *Uncaria rhynchophylla*, named as Gou-teng or Cat’s Claw, have a variety of uses in traditional herbal medicine including the treating of convulsions, numbness and hypertension (Zhou and Zhou, 2010; Ng et al., 2015; Shao et al., 2016), and in treating head ailments such as headache and dizziness. Meanwhile, *Uncaria rhynchophylla* is also commercially sold as a health food product. The consumption of *Uncaria rhynchophylla* is relatively easy today since there are so many ready-made products one can choose from, such as Cat’s Claw tea, food supplement, bark powder, and liquid extract (Pereira and Dantas, 2016). *Uncaria rhynchophylla* contains a variety of tetracyclic oxindole alkaloid structures, most notably the one that it is named after (rhynchophylline). Evidence is accumulating that the majority of pharmacological uses in *Uncaria rhynchophyll* are associated with the presence of rhynchophylline and this has have encouraged investigation of rhynchophylline as a drug candidate for neurodegenerative disorder (Xu et al., 2012; Ng et al., 2015). Recent studies have highlighted the ability of rhynchophylline to penetrate the blood-brain barrier, and the neuroprotective effects observed in CNS disease models (Lee et al., 2014). For instance, rhynchophylline was reported to act as a non-competitive antagonist of NMDA receptor and protect against glutamate-induced neuronal death (Kang et al., 2002). Additionally, rhynchophylline was able to ameliorate hippocampal synaptic dysfunction in mouse models of Alzheimer’s disease through the blockage of EphA4 signaling (Fu et al., 2014). Unfortunately, there is still little evidence about the neuroprotective effects and the underlying molecular mechanisms of rhynchophylline in models associated with PD. Herein, we introduced a cellular Parkinson’s paradigm in which toxicity was triggered by MPP^+^ in primary CGNs and found, for the first time, that rhynchophylline protected neurons via activating transcription factor MEF2D through the inhibition of GSK3β.

## Materials and Methods

### Reagents

Rhynchophylline was purchased from *Chengdu Desite Biotechnology Co. Ltd*. 3-(4,5-Dimethylthiazol-2-yl)-2,5-diphenyltetrazolium bromide (MTT), MPP^+^, Hoechst 33342 and dimethyl sulfoxide (DMSO) were obtained from Sigma (St Louis, MO, United States). Basal modified Eagle’s medium (BME) and other cell culture supplements were from Gibco (Carlsbad, CA, United States). BD Transduction Laboratories ^TM^ Purified mouse anti-MEF2D (#610774) was from BD Biosciences (BD Biosciences, San Jose, CA, United States). Antibodies against phospho-Ser473-Akt (#9271S), total Akt (#9272S), phospho-Ser9-GSK3β (#9336S), total GSK3β (#9315S) and Bax (#2772) were from Cell Signaling Technology (Beverly, MA, United States). Bcl-2 (#sc-7382) and β-actin (#sc-1616) was from Santa Cruz Biotechnology (Santa Cruz, CA, United States).

### Cultures of Primary Cerebellar Granule Neurons (CGNs)

All animal studies were approved by the Committee on the Use of Animals in Teaching or Research (No. 16-17_38-ABCT-R-GRF) at the Hong Kong Polytechnic University. All animals were purchased form the Centralized Animal Facilities (CAF) at the Hong Kong Polytechnic University. The preparation of CGNs was performed using 8-day-old Sprague-Dawley rats as we previously described (Hu et al., 2015a). Following purification, CGNs were seeded at a density of 2.0 × 10^6^ cells/ml in BME that contained 10% FBS, 25 mM KCl, 2 mM glutamine, and 1% penicillin/streptomycin mixture. Experiments were carried out after 8 days *in vitro* (DIV) because CGNs acquired a variety of features of mature neurons after 8 DIV culture.

### Examination of Cell Viability

Cell survival was measured using MTT assay as we demonstrated (Hu S. Q. et al., 2013). CGNs were pre-treated for 2 h with rhynchophylline (1, 3, 10, 30, 50 μM), and then exposed to 50 μM MPP^+^. Twenty-four or forty-eight hour after insult, CGNs were incubated for 4 h with MTT solution (0.5 mg/ml), and the generated formazan crystal was dissolved in DMSO. The absorbance was measured at 570 nm.

### Assay of Fluorescein Diacetate (FDA)/Propidium Iodide (PI) Double Staining

The live/dead cytotoxicity was examined using FDA/PI staining assay as described (Hu S. et al., 2013). Briefly, after MPP^+^ challenge, cells were incubated with FDA (5 μM) and PI (5 μM) simultaneously for 5 min, and then observed and photographed.

### MEF2 Luciferase Reporter Gene Assay Using PC12 Cells

PC12 cells were cultured in DMEM that contained 10% FBS and 1% penicillin/streptomycin mixture at 37°C. PC12 cells were transfected with a MEF2:pGreenFire1^TM^ reporter lentivector (System Biosciences, Mountain View, CA, United States). These cells were treated with rhynchophylline for 24 h, or pre-treated with rhynchophylline for 2 h prior to a 24-h incubation of MPP^+^, and then subjected to luciferase reporter gene assay.

### Short Hairpin RNA Transfection of MEF2D

ShRNA against MEF2D was designed by targeting the sequence 5′-GTAGCTCTCTGGTCACTCC-3′ (Flavell et al., 2006). Briefly, CGNs were transfected with the plasmids by using Lipofectamine 2000 as we described previously (Hu et al., 2015b). Selection medium that contained 100 μg/ml G418 was added to the cells after transfection.

### Western Blotting Analysis

Western blot analysis for exploring the signaling pathways was carried out as demonstrated (Hu et al., 2015a). After treatment, culture medium was removed and cells were homogenized with lysis buffer on ice. The extracted protein was transferred to a new microtube, and centrifuged at 12,000 rpm for 10 min. The supernatant was then separated on SDS–polyacrylamide gel and then transferred onto polyvinyldifluoride membranes. After 1–2 h of blocking (non-fat milk in Tris buffered saline with Tween20) at room temperature, these membranes were probed with primary antibodies (1:1000 dilution for p-Ser9-GSK3β, total-GSK3β, p-Ser473-Akt, total Akt, Bax, Bcl-2, MEF2D and β-actin,) overnight at 4°C, followed by 45 min incubation with secondary antibodies (1:2000 dilution) in blocking buffer. The blots were developed using a Pierce^TM^ ECL Western blotting substrate, exposed to X-ray films, and quantified by densitometric analysis using ImageJ software.

### Statistical Analysis

Data were obtained from at least three independent experiments and expressed as mean ± SEM. Multiple comparisons were assessed using one-way ANOVA, followed by the Tukey’s *post hoc* test. *P* < 0.05 was taken as significant.

## Results

### Rhynchophylline Greatly Protects Against MPP^+^-Induced Neurotoxicity in Primary CGNs

**FIGURE 1:**
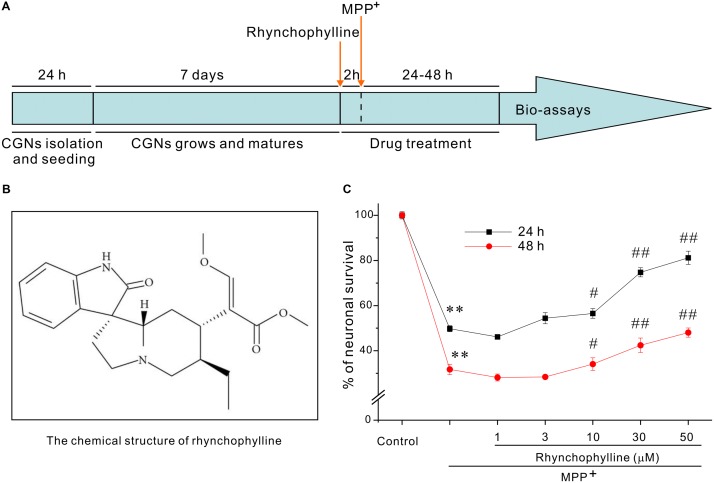
Rhynchophylline markedly protects CGNs against MPP^+^-induced neurotoxicity. **(A)** Timetable for the neuroprotection of tested compounds against MPP^+^-induced neurotoxicity in primary CGNs. **(B)** The structure of rhynchophylline. **(C)** Rhynchophylline prevents the neurotoxicity caused by MPP^+^. At 8 DIV, CGNs were pre-treated for 2 h with rhynchophylline (1, 3, 10, 30, 50 μM), and then exposed to 50 μM MPP^+^. Twenty-four or forty-eight hour after insult, CGNs were subjected to MTT assay for the evaluation of neuronal viability (*n* = 5, ^∗∗^*p* < 0.01, compared to control group; ^#^*p* < 0.05, ^##^*p* < 0.01, compared to MPP^+^ group).

The timetable for the evaluation of rhynchophylline was shown in **Figure 1A**. We had previously established a PD-related cellular model in which neurotoxicity was induced by 50 μM MPP^+^. With the use of this model, exposure of CGNs to 50 μM MPP^+^ for 24 h caused a remarkable cell death, leaving (49.76 ± 1.36) % cells alive (**Figure 1C**), while pre-treatment with rhynchophylline (**Figure 1B**, 10, 30, 50 μM) for 2 h significantly promoted neuronal viability to (56.54 ± 2.11) %, (74.75 ± 2.02) % and (81.18 ± 2.93) %, respectively. More encouragingly, rhynchophylline at 30 and 50 μM still provided neuroprotective effects even when CGNs were insulted with MPP^+^ for 48 h, significantly increasing viable cells from (31.73 ± 2.26) % to (42.46 ± 3.19) % and (48.04 ± 2.06) %, respectively. As the maximal protection was achieved at 50 μM, rhynchophylline at 50 μM was consequently used in the subsequent experiments.

### Rhynchophylline Robustly Blocks the Morphological Features of Apoptosis Caused by MPP^+^ in CGNs

**FIGURE 2:**
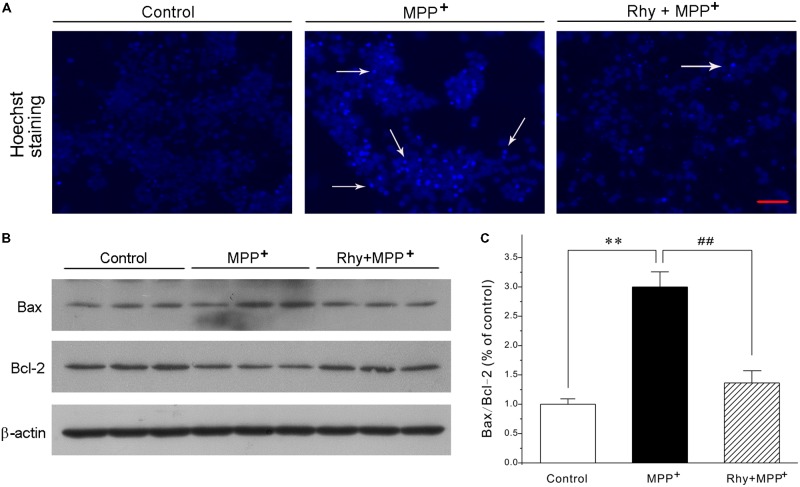
Rhynchophylline blocks the hall markers of apoptosis caused by MPP^+^ in CGNs. **(A)** CGNs were pre-treated with rhynchophylline (Rhy, 50 μM) for 2 h, then incubated with 50 μM MPP^+^. Twenty four hour later, cells were stained with Hoechst 33342 and photographed with fluorescence microscopy. The white arrows indicate apoptotic neurons. Scale bar = 50 μm. **(B)** The sister CGNs were lysed and the extracted protein was subjected to Western blotting assay using antibodies against Bcl-2, Bax and β-actin. **(C)** The statistical analysis of results obtained from **(B)** (*n* = 3, ^∗∗^*p* < 0.01, compared to control group; ^#^*p* < 0.05, ^##^*p* < 0.01, compared to MPP^+^ group).

Blue fluorescent Hoechst 33342 brightly stained the condensed chromatin of apoptotic neurons in a MPP^+^ group, but more dimly stained the normal chromatin of live neurons in the control and rhynchophylline (50 μM) plus MPP^+^ groups (**Figure 2A**). Of the known regulators of apoptosis, the best characterized parameter was the ratio of Bax to Bcl-2. As shown in **Figures 2B,C**, a shift in the Bax/Bcl-2 balance associated with MPP^+^ neurotoxicity was found in CGNs, whereas this ratio declined from (2.99 ± 0.26) % in the MPP^+^ group to (1.36 ± 0.21) % in the group of MPP^+^ plus rhynchophylline, though rhynchophylline did not affect the protein expression of Bax/Bcl-2 (data not shown).

### Rhynchophylline Markedly Activates MEF2 Transcriptional Activity at Both Basal and Pathological Circumstances

**FIGURE 3:**
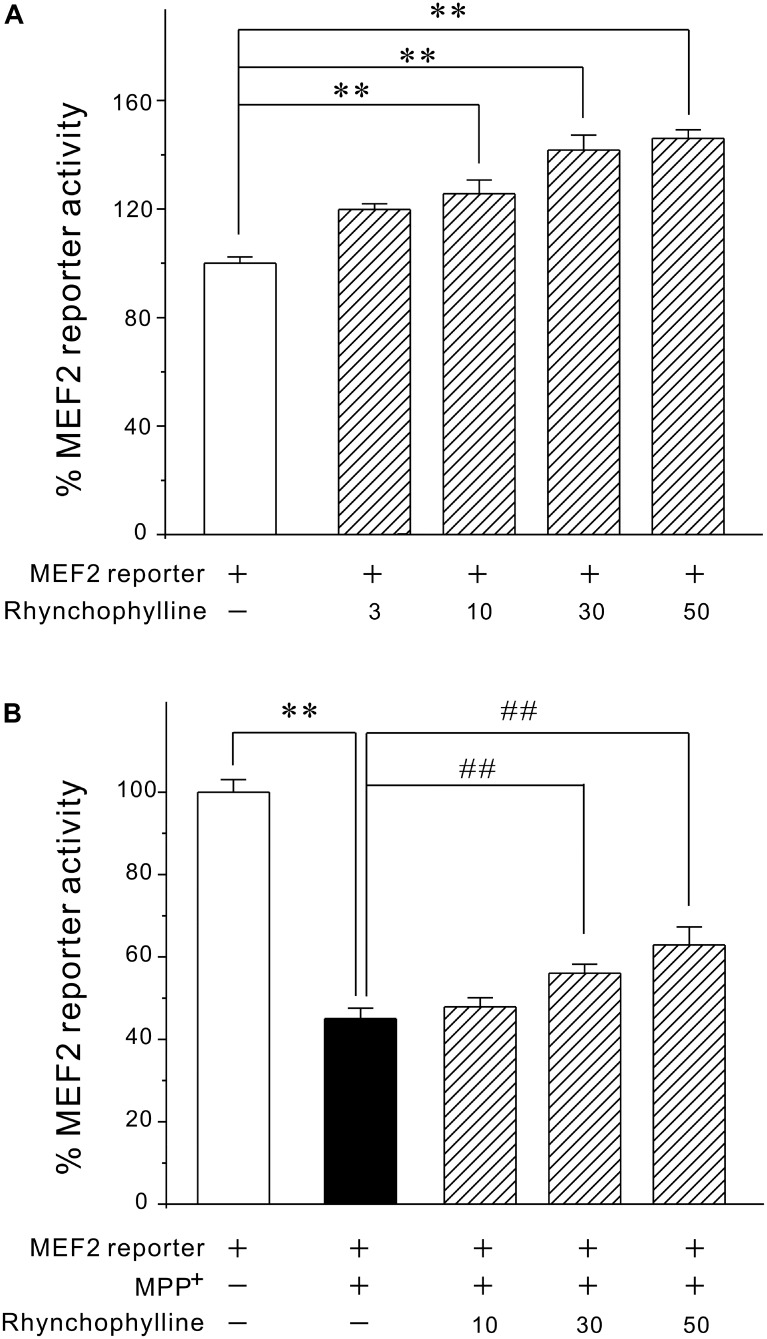
Rhynchophylline greatly activates MEF2 gene transcriptional activity in PC12 cells under both basal and pathological circumstances. **(A)** PC12 cells transfected with MEF2 luciferase reporter construct were incubated with rhynchophylline (3, 10, 30, 50 μM) for 24 h, then measured using luciferase reporter assay. **(B)** Transfected PC12 cells were pre-treated for 2 h with rhynchophylline (10, 30, 50 μM), and incubated for 24 h with 2.5 mM MPP^+^. The luciferase activity was then measured using a luminometer (*n* = 3, ^∗∗^*p* < 0.01, compared to control group. ^##^*p* < 0.01, compared to MPP^+^ group).

Since activation of MEF2 is an important mechanism underlying neuronal survival, we tested the possibility that rhynchophylline might stimulate MEF2. We transfected PC12 cells with MEF2-dependent luciferase reporter construct and incubated these cells with rhynchophylline alone for 24 h (basal circumstance), or 2 h prior to exposure of MPP^+^ challenge (pathological circumstance) for 24 h. As demonstrated in **Figure 3**, rhynchophylline at 30 and 50 μM not only promoted MEF2 luciferase activity to (141.73 ± 5.51) % and (146.02 ± 3.13) %, respectively under basal condition, but also reversed the decrease in MEF2 activity caused by MPP^+^, from (45.05 ± 2.53) % to (56.06 ± 2.20) % and (62.91 ± 4.38) %, respectively.

### Rhynchophylline Reverses the Decrease in MEF2D Levels Caused by MPP^+^ in CGNs

**FIGURE 4:**
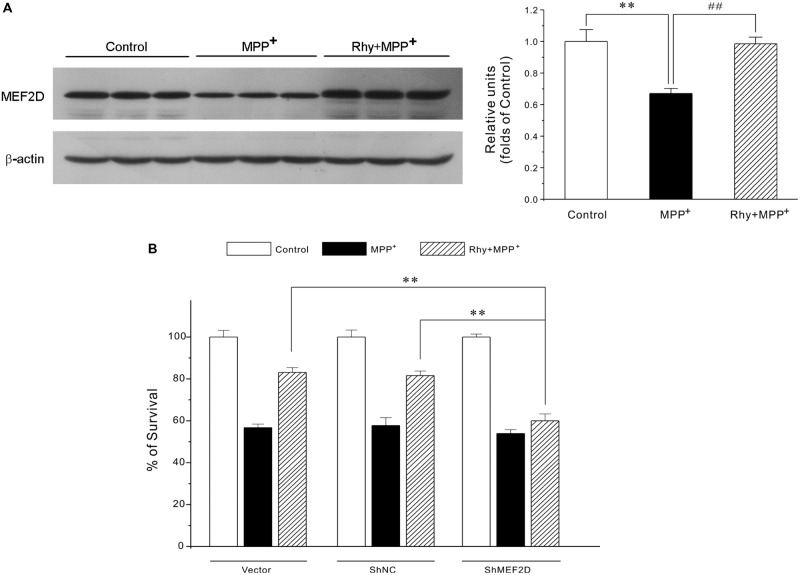
Rhynchophylline provides neuroprotection through the activation of MEF2D. **(A)** Rhynchophylline reverses the decrease in MEF2D levels caused by MPP^+^ in CGNs. CGNs were pre-treated with rhynchophylline (50 μM) for 2 h, or left untreated, then exposed to 50 μM MPP^+^ for 24 h. The cellular proteins were extracted and subjected to Western blot using MEF2D antibodies (*n* = 3, ^∗∗^*p* < 0.01, compared to control group. ^##^*p* < 0.01, compared to MPP^+^ group). **(B,C)** MEF2D gene knockdown renders rhynchophylline largely ineffective in protecting CGNs from MPP^+^-induced neurotoxicity. CGNs were transfected with pGPU6-green fluorescent protein (GFP) plasmid (vector) and pGPU6-GFP plasmid encoding GAPDH (ShNC) or MEF2D ShRNA (ShMEF2D). Forty-eight hour after transfection, cells were subjected to western blot analysis using anti-MEF2D and β-actin antibodies **(B)** After transfection, CGNs were pre-treated in the presence or absence of 50 μM Rhy for 2 h, then exposed to MPP^+^, and subjected to MTT assay for measuring cell viability **(C)** (*n* = 3, ^∗∗^*p* < 0.01, compared to the ShMEF2D group).

As demonstrated in **Figure 4A**, MPP^+^ insult caused a dramatic decrease of MEF2D in CGNs, whereas rhynchophylline (50 μM) almost completely reversed the decrease in MEF2D from (0.67 ± 0.03) to (0.98 ± 0.04). However, rhynchophylline alone did not affect the protein expression of MEF2D (data not shown).

### Gene Knockdown of MEF2D Significantly Abolishes Rhynchophylline-Mediated Neuroprotection

To further confirm the involvement of MEF2D in rhynchophylline-mediated neuroprotection, genetic silence of MEF2D using ShRNA technique was applied. This analysis showed that MEF2D gene knockdown rendered rhynchophylline largely ineffective in protecting CGNs from MPP^+^-induced neurotoxicity (**Figures 4B**).

### Rhynchophylline Down-Regulates the Protein Expression of the MEF2D Inhibitor GSK3β

**FIGURE 5:**
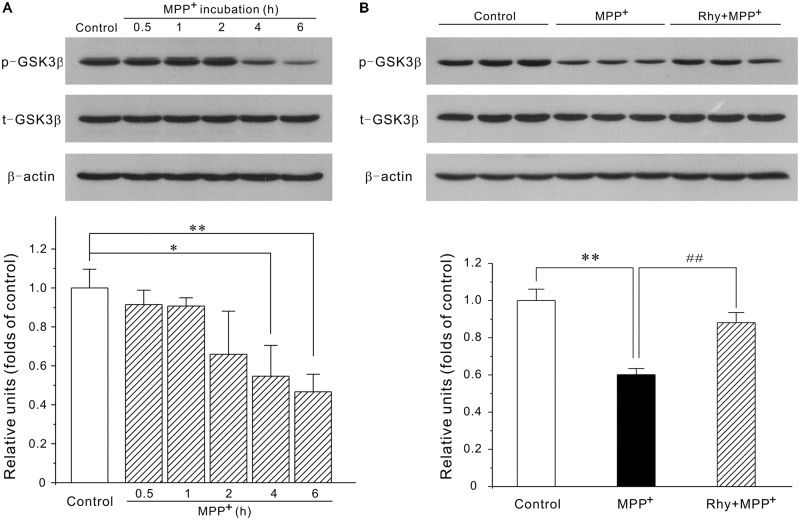
Rhynchophylline up-regulates the protein expression of p-Ser9-GSK3β. **(A)** MPP^+^ causes a time-dependent decrease in phosphorylated Ser9-GSK3β. CGNs were incubated with 50 μM MPP^+^ for an indicated time period, and the extracted protein was subjected to Western blot using p-Ser9-GSK3β and total GSK3β (*n* = 3, ^∗^*p* < 0.05, ^∗∗^*p* < 0.01, compared to control group). **(B)** Rhynchophylline prevents the decrease in the levels of p-Ser9-GSK3β caused by MPP^+^. CGNs were pre-treated with rhynchophylline (50 μM) for 2 h, or left untreated, then exposed to 50 μM MPP^+^ for 4 h, and finally subjected to Western blot (*n* = 3, ^∗∗^*p* < 0.01, compared to control group. ^##^*p* < 0.01, compared to MPP^+^ group).

Since MEF2D is a target for GSK3β, the possibility that rhynchophylline might enhance MEF2D activity through inhibiting GSK3β was herein examined. As demonstrated in **Figure 5**, MPP^+^ treatment caused a time-dependent decrease in the protein expression of phosphorylated GSK3β at Ser-9 site. GSK3β level in CGNs was down-regulated to (0.55 ± 0.15) at 4 h in response to MPP^+^, whereas rhynchophylline promoted this kinase in the phosphorylated form to (0.88 ± 0.05).

### Rhynchophylline Inhibits GSK3β in a PI3-K/Akt Dependent Manner

**FIGURE 6:**
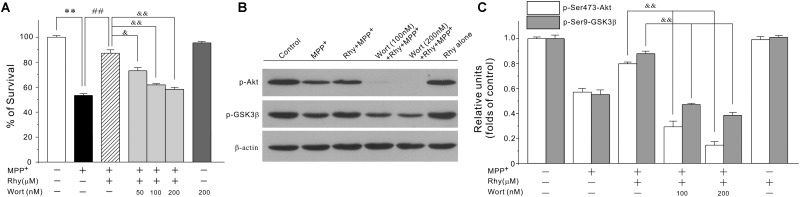
The PI3-K specific inhibitor significantly abolishes rhynchophylline-mediated neuroprotection and up-regulation of p-Ser473-Akt and p-Ser9-GSK3β. **(A)** CGNs were pre-treated with wortmannin (50, 100, 200 μM) for 2 h, then incubated with rhynchophylline (50 μM) for 2 h, and finally exposed to 50 μM MPP^+^. Twenty four hour later, cell viability was performed (*n* = 3, ^∗∗^*p* < 0.01, compared to control group; ^##^*p* < 0.01, compared to MPP^+^ group; ^&^*p* < 0.05, ^&&^*p* < 0.01, compared to the Rhy plus MPP^+^ group). **(B,C)** Cells were treated as in **(A)**. 4 h after MPP^+^ challenge, cellular protein was subjected to Western blot using p-Ser473-Akt, total Akt, p-Ser9-GSK3β and total GSK3β, respectively (*n* = 3, ^&&^*p* < 0.01, compared to the Rhy plus MPP^+^ group).

As GSK3β could be regulated by PI3-K/Akt signaling pathway, we then tested the possibility that rhynchophylline might maintain MEF2D function through the activation of PI3-K/Akt/GSK3β cascade. In our system, wortmannin, a PI3-K specific inhibitor, significantly abolished the neuroprotective effects of rhynchophylline at 50, 100, and 200 nM without affecting cell viability (**Figure 6A**). Similarly, western blot results further revealed that wortmannin largely abrogated the effects of rhynchophylline on the two protein kinases downstream of PI3-K, p-Ser473-Akt and p-Ser9-GSK3β (**Figures 6B,C**).

## Discussion

Though PD is a very common form of neurodegenerative disorder, its molecular pathology remains unclear, and the identification of intervention strategies and targets is still ongoing. The ultimate goal in PD therapy aims at arresting the relentless progression caused by the progressive loss of DA neurons. The identification of effective neuroprotectants therefore represents a promising direction for future PD therapy.

Cerebellar granule neuron are the most abundant and homogeneous neuronal types in the mammalian central nervous system. It has been well documented that these primary cells would undergo apoptosis after exposure to neurotoxins (glutamate, MPP^+^ and 6-OHDA), and that the neurotoxic molecules could trigger the activation or inactivation of similar signaling transduction pathways in CGNs as those in dopaminergic or cortical neurons (Monti et al., 2007; Li et al., 2016; Pfisterer and Khodosevich, 2017; Yu et al., 2017). In our cellular system, rhynchophylline was well characterized as an effective neuroprotectant for PD, a conclusion supported by the fact that rhynchophylline greatly prevented against MPP^+^-triggered neurotoxicity by reversing the increase in Hoechst-stained apoptotic neurons as well as the Bax/Bcl-2 ratio in primary CGNs.

Extensive studies over the past few decades have highlighted the involvement of MEF2D isoforms in the neuronal survival (Mount et al., 2013; Guo et al., 2017). Indeed, there is evidence suggesting that activation of MEF2D *in vivo* rescues DA neurons in SNpc from neurotoxicity in PD animal models (Smith et al., 2006). Very discouragingly, the use of recombinant viral approach to stimulate MEF2D transcriptional activity in these studies severely limits any further practical application. The small molecules with neuroprotective property may thereby overcome this difficulty. In the current study, we provided solid evidence that rhynchophylline exerted potent neuroprotection through the activation of the transcription factor MEF2D, a conclusion supported by the factor that rhynchophylline stimulated MEF2Dl activity under both basal and pathological conditions as well as increased the protein expression of MEF2D, and that down-regulation of MEF2D using ShRNA significantly abolished the neuroprotection of rhynchophylline. These results support the proposition that MEF2 acts as a novel pharmacological target for PD and offer molecular sights into the potential application of rhynchophylline in treating PD. And more notably, to our best knowledge, rhynchophylline is one of the very fewer naturally occurring compounds that are characterized as efficient enhancer of MEF2.

How could rhynchophylline enhance MEF2 transcriptional activity? The regulation of MEF2 activity is a complicated process. Signaling pathways and kinases associated with neuronal survival/death converge their effects, stimulatory or inhibitory, on different MEF2 isoforms. Specifically, p38 (Okamoto et al., 2000) and protein kinase A (Yin et al., 2012) primarily increase the transcriptional activity of MEF2A and MEF2C, respectively, while GSK3β (Wang et al., 2009) and CDK5 (Verdaguer et al., 2005)-mediated phosphorylation usually decrease MEF2D activity and contribute to the subsequent neuronal apoptosis. Take GSK3β, a protein kinase that is critical in the pathogenesis of neurodegenerative disease, as an example, removal of depolarization and exposure to PD-related neurotoxins (MPP^+^, 6-OHDA) over-activated GSK3β in CGNs, leading to GSK3β-dependent inhibition of MEF2D function and neuronal death (Guo et al., 2017), whereas over-expression of MEF2D mutant could be resistant to GSK3β inhibition and further protected neurons from such neurotoxicity (Wang et al., 2009). Actually, GSK3β inhibitors such as lithium have been demonstrated to inhibit the degradation of MEF2 and maintain its DNA binding ability (Linseman et al., 2003). These findings strongly suggest that MEF2D acts as a novel downstream target of GSK3β. Our findings that rhynchophylline increases the protein expression of phosphorylated-Ser9-GSK3β, a phosphorylation status that is related to lower activity of GSK3β, indicate that the stimulatory effects of rhynchophylline on MEF2D may result from the inhibition of GSK3β.

The PI3-K/Akt cascade is a pro-survival pathway critical for the development of nervous system. Inhibition of PI3-K/Akt pathway is correlated with the pathology of neurodegeneration, PD in particular. It is consequently expected that activating PI3-K/Akt may have potential protection against PD. Based on the fact that the PI3-K specific inhibitor significantly abolished the neuroprotection as well as the up-regulation of phosphorylated Ser-473-Akt and phosphorylated Ser-9–GSK3β, the inhibitory effects of rhynchophylline on GSK3β activity were possibly achieved through its activation of PI3-K/Akt signaling pathway. And more notably, we could not exclude any other possible contributing targets, such as kinases in the cytoplasm as discussed before, as MEF2D could also be directly or indirectly regulated by them. Besides, the possibility that rhynchophylline might also enhance other MEF2 isoforms will be revealed in our future projects.

It is well documented that multifactorial etiopathogenesis is closely involved in PD, and the use of multiple-drug therapy is encouraged to address the multiple pathological factors (Chong et al., 2014). However, the diversity in bioavailability may limit the clinical of drug combination. In this regard, multifunctional compound may offer greater efficacy by simultaneously targeting different sites in the brain. The identified compound rhynchophylline has been demonstrated, previously and herein, to possess multiple pharmacological activities including anti-inflammation, MEF2D enhancement and promotion of neuronal survival. This synergism may make rhynchophylline a potent candidate for further preclinical study in PD prevention.

## Conclusion

**FIGURE 7:**
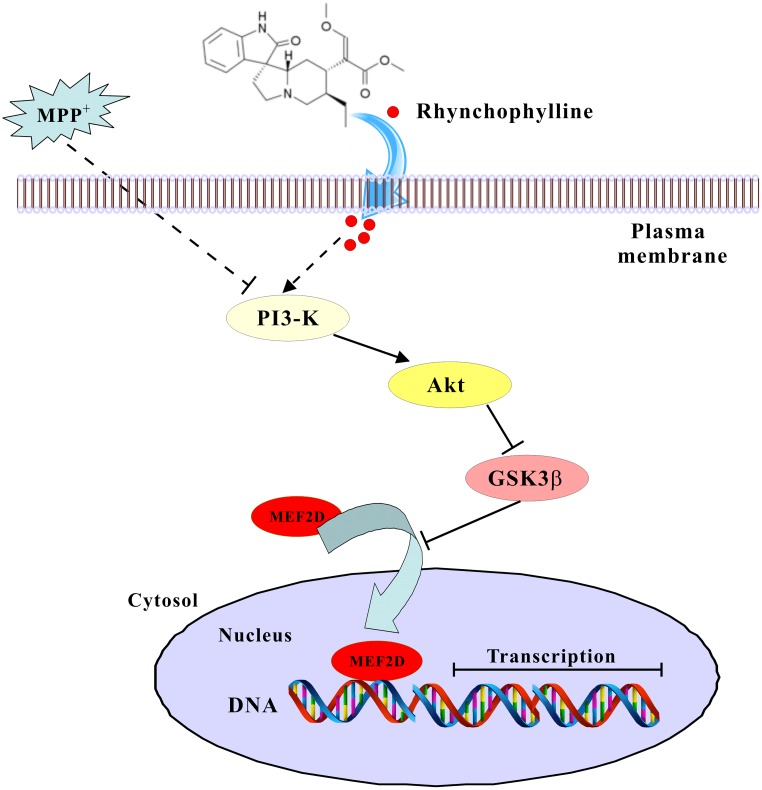
A schematic diagram shows the signaling pathway involved in the neuroprotection of rhynchophylline. In response to MPP^+^ challenge, rhynchophylline activated PI3-kinase/Akt pathway to inhibit GSK3β, leading to the nuclear translocation of MEF2D and neuronal survival.

We have provided solid evidence that rhynchophylline, a tetracyclic oxindole alkaloid from *Uncaria Rhynchophylla*, greatly protected against neurotoxicity caused by MPP^+^ in primary CGNs. These neuroprotective effects of rhynchophylline were mainly achieved through the activation of the transcription factor MEF2D, possibly via the inhibition of GSK3β (**Figure 7**). Taken together, these results suggest that rhynchophylline may be a potent candidate for further preclinical study in PD prevention.

## Author Contributions

YH designed the overall project. SH directed experiments in the various biochemical analysis including assays of MTT, FDA/PI staining, luciferase gene reporter and Western blot, and drafted the manuscript. SM and XZ performed the preparation of primary neurons. HL carried out the transfection of a MEF2:pGreenFire1^TM^ reporter lentivector in PC12 cells. YW and YH revised the manuscript. All authors read and approved the final manuscript.

## Conflict of Interest Statement

The authors declare that the research was conducted in the absence of any commercial or financial relationships that could be construed as a potential conflict of interest.

## Abbreviations

CGNs: cerebellar granule neurons
FBS: fetal bovine serum
GSK3β: glycogen synthase kinase 3β
MEF2: myocyte enhancer factor 2
MPP^+^: 1-methyl-4-phenylpyridinium
PBS: phosphate buffered saline
PD: Parkinson’s disease
PI3-K: phosphatidylinositol-3-kinase
ShRNA: short hairpin RNA
